# Refrigerated Fruit Storage Monitoring Combining Two Different Wireless Sensing Technologies: RFID and WSN

**DOI:** 10.3390/s150304781

**Published:** 2015-02-26

**Authors:** Ricardo Badia-Melis, Luis Ruiz-Garcia, Javier Garcia-Hierro, Jose I. Robla Villalba

**Affiliations:** 1 Departamento de Ingeniería Agroforestal, ETSI Agrónomos, Universidad Politécnica de Madrid, 28040 Madrid, Spain; E-Mail: luis.ruiz@upm.es; 2 Sensors Technology Laboratory, Centro Nacional de Investigaciones Metalúrgicas, Consejo Superior de Investigaciones Científicas (CENIM-CSIC), Avenida Gregorio del Amo 8, 28040 Madrid, Spain; E-Mails: j.hierro@cenim.csic.es (J.G.-H.); jrobla@cenim.csic.es (J.I.R.-V.)

**Keywords:** cold chain, logistics, post-harvest, wireless sensor networks, RFID

## Abstract

Every day, millions of tons of temperature-sensitive goods are produced, transported, stored or distributed worldwide, thus making their temperature and humidity control essential. Quality control and monitoring of goods during the cold chain is an increasing concern for producers, suppliers, logistic decision makers and consumers. In this paper we present the results of a combination of RFID and WSN devices in a set of studies performed in three commercial wholesale chambers of 1848 m^3^ with different set points and products. Up to 90 semi-passive RFID temperature loggers were installed simultaneously together with seven motes, during one week in each chamber. 3D temperature mapping charts were obtained and also the psychrometric data model from ASABE was implemented for the calculation of enthalpy changes and the absolute water content of air. Thus thank to the feedback of data, between RFID and WSN it is possible to estimate energy consumption in the cold room, water loss from the products and detect any condensation over the stored commodities.

## 1. Introduction

The production, storage, distribution and transport of cold sensitive products, occurs daily around the world. For all these products the control of temperature is essential [1]. The term “cold chain” describes the series of interdependent equipment and processes employed to ensure the temperature preservation of perishables and other temperature-controlled products from the production to the consumption end in a safe, wholesome, and good quality state [2].The inadequate management of this supply chain can reduce the acceptable quality limit of fresh fruit and vegetables (FFVs) making approximately one-third of these products be thrown away [3]. Thus, the major challenge is to ensure a continuous cold chain from producer to consumer in order to guarantee the prime condition of goods [4]. These products may be perishable items like fruit, vegetables, flowers, fish, meat and dairy products or medical products like drugs, blood, vaccines, organs, plasma and tissues. The properties of all of them are affected by temperature changes. In case of deviations from the optimal cold chain conditions, the shelf life of the sensitive products will be reduced, yet the consequences of this shelf life drop are only physically visible in the advanced stages of the supply chain in many occasions. To understand this variation is possible by a remote monitoring [5].

The quality of these products might change rapidly due to inadequate temperature and relative humidity conditions during transport and storage. Temperature variations can occur during warehousing, handling and transportation. Inadequate temperature is second on the list of factors causing foodborne illnesses, surpassed only by the presence of initial microflora in foods [6]. The U.N. FAO estimates that each year, approximately one-third of all food produced for human consumption in the world is lost or wasted [7]. Thus, studying and analyzing temperature gradient data inside refrigeration rooms, containers and trucks is a primary concern for the industry. Any temperature disturbance can undermine the efforts of the whole chain [8]. 

It is not easy to maintain appropriate conditions over the whole chain, and negligence or mishandling in the logistics of perishable food products is very common, including goods poorly or excessive cooled. Jedermann [9] reported many examples where the inadequate management during temperature control usually leads to losses in the food chain (post-harvest, distribution and at home).

Apart from temperature, water loss is one of the main causes of deterioration that reduces the marketability of fresh fruits and vegetables. Transpiration is the loss of moisture from living tissues. Most weight loss of stored fruit is caused by this process. Relative humidity (RH), temperature (T) of the product, temperature of the surrounding atmosphere, and air velocity all affect the amount of water lost from perishable food products. Free water or condensation is also a problem as it encourages microbial infection and growth, and it can also reduce the strength of packaging materials [10,11]. 

Wireless technologies have been considered key technological enablers, whereby intelligent tags are attached to an item, within a network with wireless links, which can transmit some physical parameters such as temperature or humidity in addition with other information like position or movement [12]. 

Some studies have demonstrated that a proper control over the cold chain using RFID loggers can lead to reduced waste of products by using the “First Expiring First Out”, rather than the classical “First In First Out” paradigm, and also have implemented prediction temperature methods in order to improve the estimation of shelf life [13]. RFID tags are not limited to identification or temperature and RH reading tasks, and recent advancements have produced RFID units able to identify volatile chemicals such as ethylene or ammonia [14].

Specialized Wireless Sensor Network (WSN) monitoring devices can revolutionize the shipping and handling of a wide range of perishable products by providing suppliers and distributors with continuous and accurate readings throughout the distribution process. In this framework, WSN was developed as a very promising technology due to its low energy consumption and advanced networking capabilities. The potential for monitoring with both technologies, WSN and RFID, has been suggested by several studies; however there is still a lack of implementation and experimentation in real environments, and no study combines the advantages of using both at the same time [15,16,17,18,19]. 

The paper is focused on the analyses of the performance of semi-passive RFID loggers and WSN motes, in order to enable an economical solution for the spatial profiling of refrigerated chambers, with a high number of loggers. The aim of this work is to demonstrate the compatibility and feedback in a combination of the RFID and WSN in order to improve the refrigerated storage of perishable food products. The authors also want to provide some recommendations to improve current wireless devices and some management protocols or rules for the usage of the cold storage rooms.

## 2. Materials and Methods

### 2.1. Refrigerated Chamber

Experiments were performed in three commercial wholesale refrigerated stores, numbered 11, 29 and 40 and each of which has a volume at of 26 m × 6 m × 12 m = 1848 m^3^, with an on/off glycol cooling system and insulated walls built of foam sandwiched between two layers of corrugated plate (total wall thickness is 0.16 m). The set point experimentation times were different in each one (see Table 1). Each chamber has a common pre-chamber space where a devoted sensor is placed. Therefore, two different ambient conditions occur: pre-chamber (right outside the chamber) and the inside chamber with a well-known set point (see Table 1).

**Table 1:** Experimental conditions.

Cold Store Number	Set Point (°C)	Experimentation Time (days)	Dates
11	8	13	7–19 July
29	7	8	20–27 July
40	14	4	28–31 July

**Figure 1:**
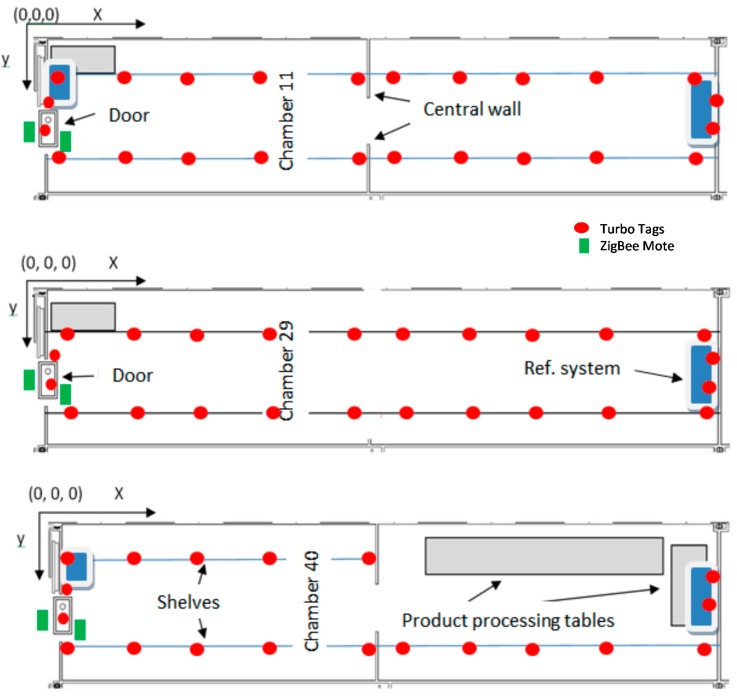
Scheme of the cold rooms and sensor distribution.

Chamber number 11 was divided into two equal size sections by a wall with a door that remained open all the time. The dimensions of the door were the same as the main door to the exterior of the whole room, *i.e*., it was big enough to allow a forklift with a big pallet to pass. The room was provided with two cooling systems, one per each section of the chamber. Inside were two shelves on either side of the room with three heights, where the products were stored. When the temperature of the room reached 8 °C the refrigerator was triggered to cool down to 6.5 °C. A scheme of the chambers can be seen in Figure 1.

Chamber number 29 had identical dimensions but with no separating wall in the middle, and the temperature was set at 7 °C and dropped to 5.5 °C. Both these rooms were designated to store mostly citric fruits and different varieties of nuts.

The last chamber (number 40) was divided into two sections separated by a wall with a semiautomatic blind door; the first section was designated for the storage of vegetables and the other section was designated for fruit and vegetable packaging.

### 2.2. WSN Motes

Two ZigBee/IEEE 802.15.4 motes (transmitters) and one base station (receiver) were used. One mote was installed outside the chamber, close to the door and the other one inside, at the other side of the wall. Sampling rate was set to 180 s. 

These motes were manufactured by Crossbow^®^ (Milpitas, CA, USA ) and they have a microcontroller board (IRIS, Milpitas, CA, USA) together with an independent transducer board (MTS400, Andover, MA, USA) attached by means of a 52 pin connector.Its processor & radio platform is a XM2110CA, based on the Atmel ATmega1281. The RF power was configured to 3 dBm (60% higher than previous MICA Motes). Power was supplied by two AA lithium batteries.

The MTS400 board hosts a variety of sensors: temperature and relative humidity (Sensirion SHT, Staefa, Switzerland), barometric pressure and temperature (Intersema MS5534B, Hampton, VA, USA), light intensity (TAOS TSL2550D, Plano, TX, USA) and a two-axis accelerometer (ADXL202JE, Norwood, MA, USA). A laptop computer is used as the receiver, and it communicates with the nodes through a Micaz mounted on the MIB520 ZigBee/USB gateway board; this device also provides a USB programming interface. In this study only the data from the Sensirion and Intersema units was used.

The Sensirion SHT is a single-chip relative humidity and temperature multi-sensor module that delivers a calibrated digital output. Each SHT is individually calibrated in a precision humidity chamber. The calibration coefficients are programmed into the One Time Programmable (OTP) memory. These coefficients are used internally during measurements to calibrate the signals from the sensors.

For temperatures significantly different from 25 °C, and according to the manufacturer recommends performing humidity and temperature compensation, using Equations (1) and (2):

*RH_linear_* = (*−*4) + 0.0405 × *SO_RH_* + (*−*2.8 × 10*^−^*^6^) × *SO_RH_^2^* (1)

where *SO_RH_* = Sensor Output Relative Humidity.

*RH_true_* = (*T °*C − 25) × (0.01 + 0.00008 × *SO_RH_*) + *RH_linear_* (2)

The MS5534B is a SMD-hybrid device including a piezoresistive pressure sensor and an ADC-Interface IC. It provides a 16 bit data word from a pressure and temperature (−40 to +125 °C) dependent voltage. Additionally the module contains six readable coefficients for a highly accurate software calibration of the sensor. MS5534B is a low power, low voltage device with automatic power down (ON/OFF) switching. A 3-wire interface is used for all communications with a microcontroller. Sensor packaging options are plastic or metal caps. 

### 2.3. Semi-Passive RFID Tags

RFID tags can be active, passive, or semi-passive. Passive and semi-passive RFID send their data by reflection or modulation of the electromagnetic field that was emitted by the reader. The battery of semi-passive RFID is only used to power the sensor and recording logic [1]. In this study we used semi-passive tags. 

Up to 90 RFID semi-passive loggers were installed in each chamber, the tags are distributed in four layers or heights; each layer is as shown in Figure 1. The tags were manufactured by Sealed Air (New York, NJ, USA), and two different types were used, a Turbo Tag T700 (−30 °C to +55 °C) and the T702-B (−70 °C to +80 °C). Each tag operates in the 13.56 (ISO 15693-3 compliant) MHz band, has the size of a credit card, is accurate to within ±0.5 °C, and both have KSW Microtec (Dresden, Germany) temperature sensors and can store up to 702 temperature measurements.

### 2.4. Data Analysis

In the particular case of the WSN system, since its task consists of sending data to a gateway in a continuous flow, a specialized Matlab program has been developed for assessing the percentage of lost packets (%) in transmission, by means of computing the number of multiple sending failures for a given sample rate (SR). A multiple failure of “m” messages occurs whenever the elapsed time between two messages lies between 1.5 × m × SR and 2.5 × m × SR. For example, with a sample rate of 11 s, a single failure (m = 1) occurs whenever the time period between consecutives packets is longer than 16.5 s (1.5 × 1 × 11) and shorter than 27.5 s (2.5 × 1 × 11). The total number of lost packets is computed based on the frequency of each failure type. Accordingly, the total percentage of lost packets is calculated as the ratio between the total number of lost packets and the number of sent packets. 

The standard error (SE) associated to the ratio of lost packets is computed based on a binomial distribution as expressed in Equation (3), where n is the total number of packets sent, and p is the ratio of lost packets in the experiment:

(3) $$SE=\sqrt{\frac{p(1-p)}{n}}$$

A second dedicated Matlab code was used for plotting 3D temperature gradients. This program makes use of linear spatial interpolation in order to obtain 3D representations of normalized temperatures and variances inside the chambers. Due to the significant variation of external conditions in the Spanish summer, temperature analysis make use of a normalized temperature difference (∆Tn), which is computed with respect to the set point and to the outside temperatures (see Equation (4)). This value gives a normalized measure with respect to the varying ambient conditions of the experiments:

(4) $$\Delta T_{n}=\sqrt{\frac{T_{m}-T_{s}}{T_{e}-T_{s}}}$$

where ∆*T*_n_ (dimensionless) stands for the normalized temperature difference, *T*_m_ is the average temperature value (°C) of each RFID logger, *T*_s_ is the temperature set point value (°C), and *T*_e_ refers to the average outside temperature (°C).

Finally, a 3D plot of indoor temperature variance with regard to outdoor temperature variance is recorded in order to represent the indoor temperature variability corrected with regard to changes in ambient experimental conditions (see Equation (5)):

(5) $$V_{n}=\frac{V_{i}}{V_{e}}$$

where *V*_n_ is the normalized indoor variance for experiment, *V*_i_ is the indoor temperature variance of each logger, and *V*_e_ is the outside variance. The indoor variability is expected to be lower than the pre-chamber variability, this value will give us an estimation of the differences in the temperatures in the two ambient; the closer to the unit the normalized indoor variance is, the closer the two variances are. This can provide an idea of the influence of the outdoor temperatures without taking into account the set point, therefore it is possible to evaluate the transit of the staff and the isolation of the cold room itself, while in the normalized temperature difference the cooling unit appears as a factor in the equation. The complete set of data is included for each experiment:

### 2.5. Psychrometric Data 

The ASAE D271.2 standard, defined in April 1979 and reviewed in 2006, is used for computing the psychrometric properties of air [20]. Equations (6)–(8) and Table 2 enable the calculation of all psychrometric data of air whenever two independent psychrometric properties of an air-water vapor mixture are known in addition to the atmospheric pressure:

(6) $$Ps=R*e^{\frac{A+B*T+C*T^{2}+D*T^{3}+E*T^{4}}{F*T-G*T^{2}}}$$

273.16 K ≤ T ≤ 533.16 K. *T* = Temperature (K), *Ps* = Saturation vapour pressure (Pa) [20].

(7) $$Pv=Ps\frac{RH}{100}$$

*Pv* = Vapor pressure (Pa) [20].

(8) $$H=\frac{0.6219*Pv}{Patm-Pv}$$

*H* = Absolute humidity (g/kg dry air), *P_atm_* = Atmospheric pressure (Pa) [20].

**Table 2:** Coefficients used to compute the psychrometric data, according to Equation (7) [20].

R = 22,105,649.25	D = 0.12558 × 10^−3^
A = −27,405.526	E = −0.48502 × 10^−7^
B = 97.5413	F = 4.34903
C = −0.146244	G = 0.39381 × 10^−2^

## 3. Results and Discussion

The presentation of the results in this work is divided into three parts. The first is the percentage of lost packets in the WSN transmission, which is key knowledge since is telling how much information is lost in translation, and it is saying that probably there should be a better transmission, the reasons are either a reading range problem, volumes of data are too big or there is some physical limitation, e.g. a massive amount of loggers, as Ruiz-Garcia & Lunadei address in their article [1], there are certain challenges and limitations that hinder a perfect data flow. That is another reason why RFID shows good complementarity with WSN, and the data loss can be addressed with the RFID tags.

The second part is about the temperature distribution, and as it was only possible to carry out this measurement due to the high volume of data generated from the RFID tags; it allows creating a uniform mesh of temperature nodes with many data points which is necessary for the 3D representation, in this case this big volume was not a limitation, but when there is a good understanding of the environment, certain spots of the cold room can be monitored with temperature estimation methods and bypass the actual sensors, as was successfully shown by other authors [21,22,23].

The third part is the psychrometric representation, where both the humidity sensor like the temperature sensor (in the WSN nodes) are gathered in the Sensirion SHT unit. This graphics are meant to compare the air inside and outside the cold rooms close to the door.

### 3.1. Percentage of Lost Packets

The 3 dBm of RF power used by the motes was enough to enable sending data outside the chamber. More than 98% of the packets sent were able to cross the walls (see Table 3). Only 1.25% of signals were lost in the worst case (Chamber 11). In previous experiments [24] under very similar conditions, the average of lost packages was 1.92% ± 0.07%, in that case the gateway was located in the same room as the mote and in our experiment one of the motes is across the isolating door of the room for better performance.

**Table 3:** Percentage of data lost packets and standard error during the experiments.

Cold Store Number	Mote Inside (Data)	Mote Outside (Data)
11	0.26 ± 0.01 (6117)	1.25 ± 0.01 (6007)
29	0.24 ± 0.02 (3244)	0.89 ± 0.02 (3223)
40	0.21 ± 0.02 (1846)	0.70 ± 0.02 (1826)

### 3.2. 3D Plots

**Figure 2:**
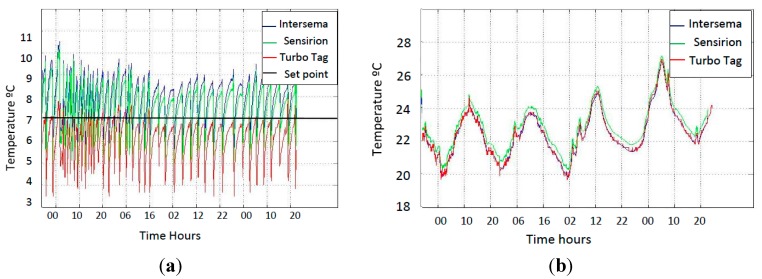
(**a**) Absolute temperature inside chamber 29; (**b**) Absolute temperature pre-chamber 29.

**Figure 3:**
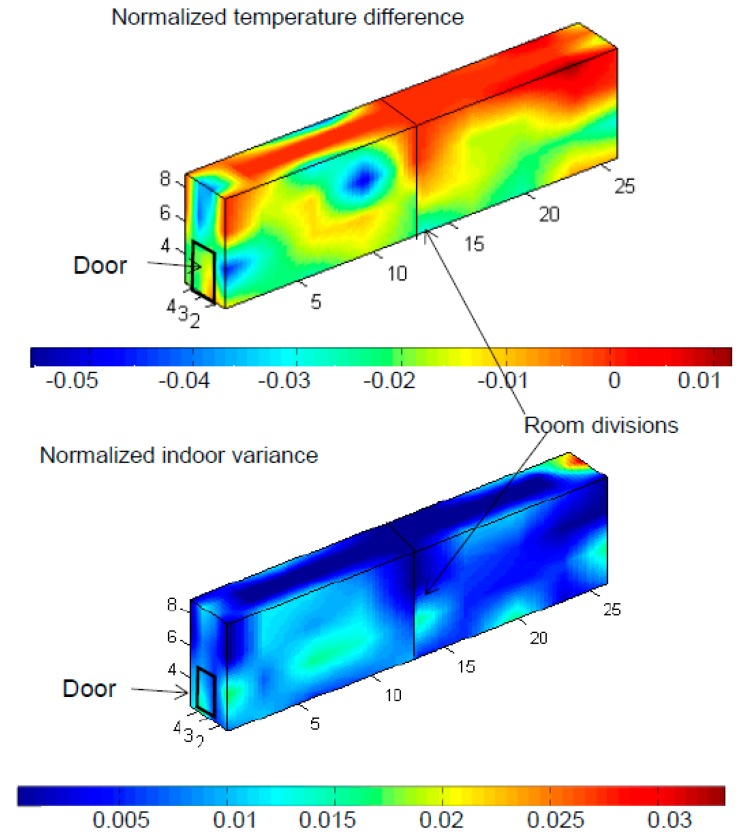
3D plot of Normalized Temperature Difference and Indoor Variance in Chamber 11.

**Figure 4:**
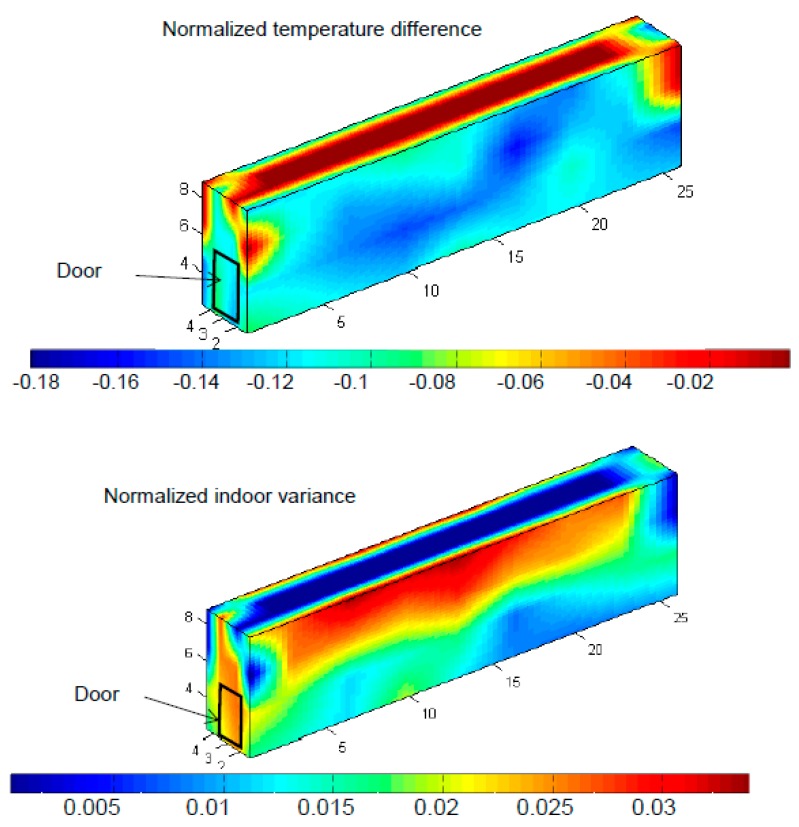
3D plot of Normalized Temperature Difference and Indoor Variance in Chamber 29.

Before starting with the 3D views, it is important to take a look on the absolute temperatures, which will give the reader a better understanding of the following Figure 3 and Figure 4. Figure 2a shows the cooling cycles inside the cold room, recorded by the sensors in the WSN mote and the RFID tag located near the mote. Accordingly with the normalized temperature one can appreciate that the temperature from the Turbo tag rarely surpasses the set point, and that gives a negative normalized temperature. In Figure 2b the day and night cycles are shown and the differences between the maximum and minimum values are around 5 °C and are bigger than inside the room (around 3 °C), which gives a normalized indoor variance between 0 and 1.

Figure 3 and Figure 4 show a summary of the 3D views corresponding to temperature behavior for Chambers 11 and 29. Both the normalized indoor variance (Δ*T*_n_) and normalized temperature variance are represented. In both cases mean temperatures were close to the set point, which means almost 0 normalized indoor variance (Δ*T*_n_ = 0). The highest differences in room 11 were registered by the sensors located in the ceiling, where in the back part of the room it can reach a normalized temperature difference (NTD) of 0.01 points and in a spot in the front part it goes to −0.05 points. In Figure 3, in Chamber 11 one can appreciate how the temperatures are closer to the set point in the back part of the room with an average of −0.01 than in the front part with a lower average −0.03, the front part presents the most different temperature from the set point despite the fact the two parts of the room each have a cooling system, so it would be justified by the influence of the transit of people going in and out of the room to cause the opening and closing of the door repeated times it makes the refrigeration system work harder and makes that the NTD goes below the set point, reaching at some points less that −0.05 units. The NTD corresponds with the normalized indoor variance (NIV) where one can appreciate as well the separation by the wall and how the variance values are higher where the door on the outside is located, with an average in NIV of 0.01.

Room 11 shows a NIV always below the unit which means that the indoor variance is always lower than the outside one; this is justified by the big variability of temperatures during the Spanish summer, and the controlled temperature inside the cold rooms. The temperature in Chamber 11 was horizontally less homogeneous (having more or less an average NIV of around 0.01 in the entire room with no differentiated layers) than in Chamber 29, which has different NIV levels each with a homogeneous NIV such as 0.03, 0.02 and 0.015, which can be justified by the existence of an intermediate wall that divides the refrigerated chamber into two zones, while Chamber 29 is less homogeneous vertically having the perfectly differentiated layers mentioned above, which is justified because the Chamber 29 is only provided with one cooling unit for the same space than room 11 which is provided with two units, one per each part of the room.

Room 29 as is said (Figure 4), is not provided by a separation wall, which makes the air flow along the space without interruptions, but with the characteristic that it has to cool down a space double the size of Chamber 11. NTD gets closer to the set point value close to the ceiling of the room than in the rest of it, reaching in some spots almost 0, but always below the set value, while in the rest of the room it has an average NTD of −0.13. 

### 3.3. Psychrometry

**Figure 5:**
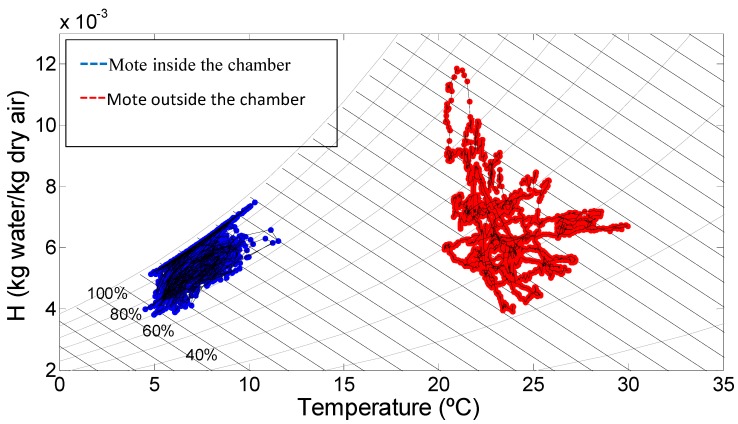
Psychrometric chart in Chamber 29.

**Figure 6:**
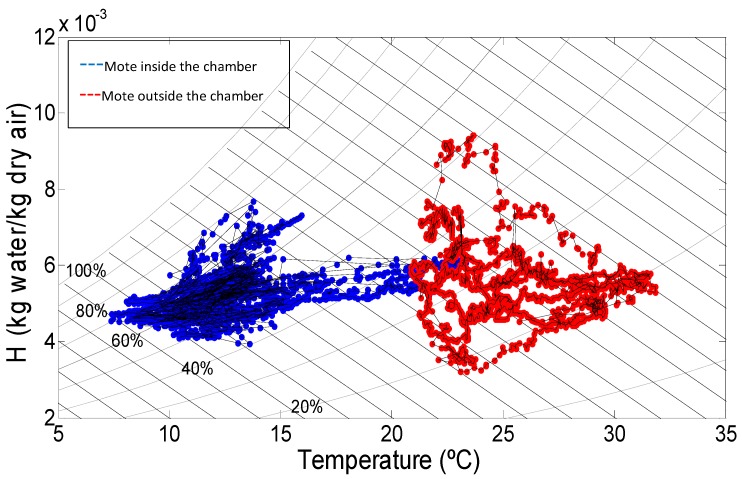
Psychrometric chart in Chamber 40.

The absolute humidity of the air inside the chambers was calculated, based on the ASAE standard D271.2 (1979) [20], using the data recorded during experiments. Psychrometric charts for two chambers (numbers 29 and 40) are included in Figure 5 and Figure 6, which illustrate the evolution of air absolute humidity (H, kg of water/kg of dry air) related to the T (°C). The blue cloud corresponds to the mote inside the chamber while the red one refers to the mote located outside (pre-chamber). Door openings created an increase in T (°C) and H (kg of water/kg of dry air), which then returns to normal again once the door is closed. During the rest of the time, it is also possible to detect the interaction between air properties and the product; with the cycles of cooling, variations in the absolute humidity can be estimated: condensation over the products (as loss of absolute humidity), or water evaporation (as an increase in absolute air humidity). The diagram corresponding to room 11 is similar to that of room 29, which is why is not presented in this article.

In Figure 5, corresponding to Chamber 29, an important difference of temperatures between inside and outside the room is appreciated, as the blue cloud in many occasions reaches a RH of 100%, water saturation in the air, and this cloud of dots is always higher in RH lines than the cloud corresponding to the outside. One can appreciate differentiated paths moving up and down the RH lines, corresponding to a vapor condensation and increase of the temperature due to the door opening, when the air with different enthalpies is mixing. Both the inside node and the outside node present very similar lower H (equal to 4 kg water/kg dry air), while the upper H reaches more than 12 kg water/kg dry air in the outside node, and inside does not go beyond 8 kg water/kg dry air. There is no exchange of air flow at any moment; the air masses are clearly separated. The cloud is more compact in the interior of the room than in the exterior and it remains around 5 to 10 °C, while outside it has a wider temperature interval (from 20 to 30 °C) and with the same absolute humidity, it stays below in RH.

In this case (Figure 6) the air inside has an H oscillating in a smaller range (between 4 and 8 kg water/kg dry air) than in the outside (between 3 and 10 kg water/kg dry air), and a much wider range of temperatures than the other two chambers (Figure 5) because the room was open during long periods of time; it even reaches 23 °C, which is a regular temperature registered in the node outside. The blue cloud (inside) shows horizontal movements corresponding to an increase in temperature due to the door opening. The outside cloud contains wide cycles that correspond to an opening and closing of the door and/or changes between day and night, since the pre chamber area is directly influenced by the heat of the sun. In contrast to Chamber 29, it is appreciated how the temperatures are equalized in the middle of the image due to the fact the door stays open for a long time, which is causing the mixing of the air and heat exchange, and a temperature and humidity gradient is also appreciated inside the chamber, in some occasions the RH reaches 80% whereas outside it never goes above 55% despite the door usually being open.

The use of a psychrometric equation provides very valuable information regarding air fluxes and water transport phenomena from the product to the air. Using two sensors of just one single mote it is possible to obtain such information in real time, and one can conclude by observing Figure 4 and Figure 5, both from Chamber 29, that the data obtained are complementary, since it is not possible to deduce that there is no exchange of air flow at the door by observing Figure 4 alone. In addition, the temperature registered by the inside node near to the door is not representative of the entire room; therefore the RFID tags are completing the data pool for the subsequent analysis. The continuous data flow coming from the nodes can be accessed at any moment; the nodes are located in critical spots (like the doors). This information is an indicator of the surrounding area, which can be studied in detail thanks to the data obtained from the temperature tags.

## 4. Conclusions

In this paper a practical demonstration in a real scenario of the complementarity of RFID semi passive tags and the WSN is shown. The performance of the RFID tags and the WSN nodes was positive overall, the data losses was acceptable, small enough to allow one to analyze the parameters in order to create temperature distribution maps and psychrometric diagrams, but this set up is only possible in a testing phase. Since such a massive quantity of RFID tags is not possible in this scenario in the way those were positioned, it means an *ad hoc* version of this system should be developed for each case. The relative low cost of the RFID loggers allows dense implementations that provide accurate information about temperature gradients inside the chambers. The amount of data obtained from all the tags is manageable with a proper program such as devoted programs developed in Matlab. In the other hand extracting the data from a big number of these units in the cold rooms that are subject of study was a difficult task to accomplish, due to the location and the main limitations of these loggers which are their reading range and sensing capabilities. 

Some of the loggers could be substituted by certain temperature estimation methods, but although these techniques are demonstrated to be reliable, other loggers located in critical spots should not be removed since there are changing conditions that cannot be predicted, such as the movement of the pallets inside the room.

Further experiments can provide a closer implementation of the two technologies, by developing a complex system comprising WSN nodes and RFID units. The benefits of this implementation are shown in this work, on the one hand WSN presents a long reading range thanks to the point to point (PtP) data transmission and a constant information flow, while in the other hand RFID lacks a PtP chain and also the tags used in the present experiments do not send information unless requested by the reader. The high price of the sensor nodes makes a dense node mesh difficult to afford, but this weakness could be resolved by using cheaper tags, and also the RFID tags are more manageable and robust due to their encapsulation.

After a detailed analysis of the data gathered in these experiments, and the experience achieved using the devices that are provided with the sensors used, the authors would like to recommend some improvements and changes to be made in the construction of the sensors. The reading range should be addressed; long range reading fixed readers or handhelds should be implemented and enhanced so communication of large amounts of data can be done with commercial RFID temperature tags. Also the range of sensing can be improved, some prototypes like the named in the introduction of this document could be turned in commercial units, as shown in this article, the use of pressure and humidity is a useful tool to see the air exchange in critical points such as the door of the cold room. As demonstrated in the experiment, WSN nodes can provide information that can be essential to detect breaks in the cold chain, nonetheless WSN nodes lack robustness, these devices are always weak and the electronics are very exposed to any possible damage, so manufacturers should work on motes able to support friction, impacts and adverse climatological conditions. Maybe some kind of hybrid unit creating a RFID sensor network, where the lightness, robustness and price from RFID is combined with the extended reading range PtP and diversity of sensors characteristic from the WSN units.

In the other hand, the authors want to provide some chamber design rules and management protocols that can save energy consumption costs in the cold room and help to extend the freshness of the cargo. As seen in the Chamber 11 the back part of it is closer to the set point than the front which is connected with the pre-chamber through a door, therefore, the most sensitive goods should be placed in the back part. In Chamber 40, a big waste of energy was noted because the outdoor and indoor air is being mixed constantly, hence the stored vegetables should be placed in the back part, and the processing tables moved to the front part. Finally an influencing factor that affects all the rooms is the opening and closing of the door, so a cooled pre-chamber should be studied; the actual pre-chamber is a corridor connected with the exterior with no isolation, and this has a certain negative effect on the temperatures when there is transit of the staff.
